# Industry 4.0 Lean Shopfloor Management Characterization Using EEG Sensors and Deep Learning

**DOI:** 10.3390/s20102860

**Published:** 2020-05-18

**Authors:** Daniel Schmidt, Javier Villalba Diez, Joaquín Ordieres-Meré, Roman Gevers, Joerg Schwiep, Martin Molina

**Affiliations:** 1 Department of Business Intelligence, Escuela Técnica Superior de Ingenieros Industriales, Universidad Politécnica de Madrid, 28006 Madrid, Spain; j.ordieres@upm.es; 2 Matthews International GmbH, Gutenbergstraße 1-3, 48691 Vreden, Germany; roman.gevers@saueressig.de (R.G.); joerg.schwiep@saueressig.de (J.S.); 3 Hochschule Heilbronn, Fakultät Management und Vertrieb, Campus Schwäbisch Hall, 74523 Schwäbisch Hall, Germany; 4 Department of Artificial Intelligence, Escuela Técnica Superior de Ingenieros Informáticos, Universidad Politécnica de Madrid, 28660 Boadilla del Monte, Madrid, Spain; martin.molina@upm.es

**Keywords:** EEG sensors, manufacturing systems, shopfloor management, machine learning, deep learning

## Abstract

Achieving the shift towards Industry 4.0 is only feasible through the active integration of the shopfloor into the transformation process. Several shopfloor management (SM) systems can aid this conversion. They form two major factions. The first includes methodologies such as Balanced Scorecard (BSC). A defining feature is rigid structures to fixate on pre-defined goals. Other SM strategies instead concentrate on continuous improvement by giving directions. An example of this group is the “HOSHIN KANRI TREE” (HKT). One way of analyzing the dissimilarities, the advantages and disadvantages of these groups, is to examine the neurological patterns of workers as they are applying these. This paper aims to achieve this evaluation through non-invasive electroencephalography (EEG) sensors, which capture the electrical activity of the brain. A deep learning (DL) soft sensor is used to classify the recorded data with an accuracy of 96.5%. Through this result and an analysis using the correlations of the EEG signals, it has been possible to detect relevant characteristics and differences in the brain’s activity. In conclusion, these findings are expected to help assess SM systems and give guidance to Industry 4.0 leaders.

## 1. Introduction

The concept of Industry 4.0 tries to tackle many of the forthcoming challenges that get faced by business leaders in the 21st century [1,2]. Some may see it as focused on technological solutions for higher automation enabled by digitalization and the resulting possibilities [3]. Yet the full potential is only achievable through the holistic perspective of a sociotechnical system [4,5]. This stands in stark contrast to the extreme technical view of Computer Integrated Manufacturing (CIM) [6]. In the sociotechnical system, the technology and the workers are both viewed as interconnected parts. Because of this, placing a prime focus on the complex interaction between these two groups is necessary [7]. This presupposes that organizations will change old structures and adapt to the approaching demands [8]. The challenge presented here, which is closely linked to the research question, is whether artificial intelligence is capable of providing future leaders with the tools necessary to understand the complex dynamics that occur in these Industry 4.0 environments.

**Figure 1:**
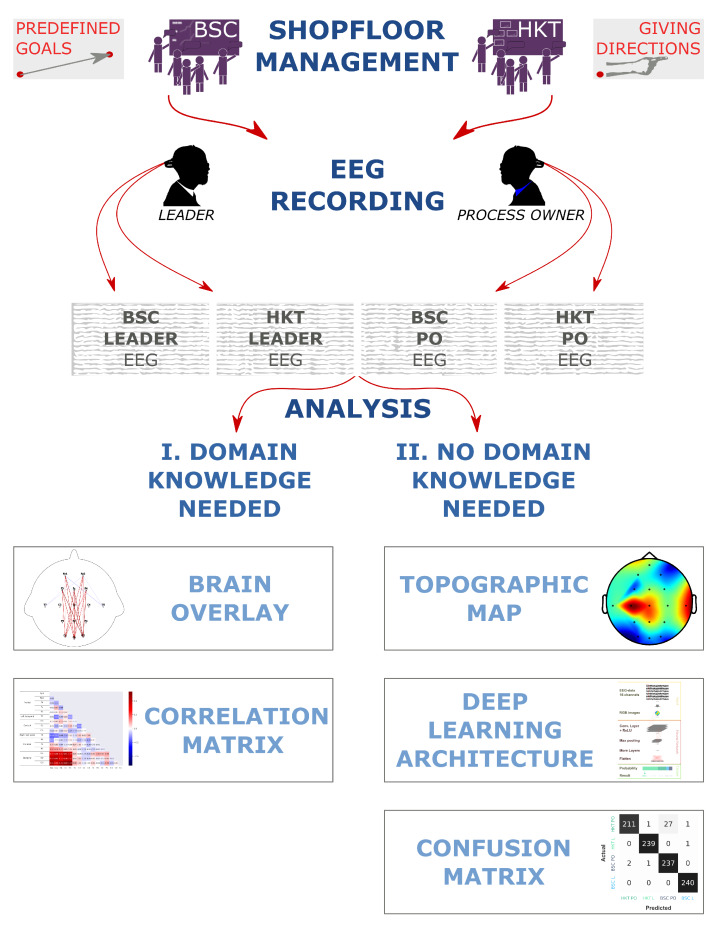
Graphical Abstract.

The purpose of this work is to provide Industry 4.0 leaders with a better understanding of Lean Shopfloor Management (SM) methods through artificial intelligence techniques applied to information collected from portable devices that provide an electroencephalographic (EEG) signal. For this the initial hypothesis is that deep learning (DL) algorithms are capable of characterizing and discerning between different types of behavior, once the EEG signal has been properly treated. This is shown in the graphical abstract of the paper depicted in Figure 1. More specifically, the work aims to offer insights into the two major categories of SM systems. This is done through the study of the neurological activity of process owners and their leaders performing either method. Non-invasive EEG sensors capture the electrical activity of the brain. A DL soft sensor is used to categorize the data. If the hypothesis is correct, then this would confirm that distinct contrasts in the brain activity during the conduction of the different SM systems exist. Furthermore, the correlations of the sensor channels are compared. These correspond to brain regions and show existing differences.

In the following the structure of the paper is described. Section 2 provides a background to the lean SM methodologies which presents fundamental preliminary concepts to the comprehensive understanding of the presented content in the following sections of this work. Section 3 provides the neurophysiological background in the context of SM, necessary to understand the EEG classification performed by the DL algorithms. Section 4 demonstrates the used materials and methods. The results attained are discussed in Section 5 and the implications in Section 6 should be able to help judge disparate SM systems and give guidance to Industry 4.0 leaders.

## 2. Background

In order to achieve this goal, following points have received close attention. These and the rest of this section are, apart from the further description of the paper, taken from the conference paper The HOSHIN KANRI TREE. Cross-Plant Lean Shopfloor Management [9].

The points are:

- the need to sustainably empower the workforce (Learning Factory) (LF) as indicated by Narkhede et al. [10],
- the need to develop an autonomous and intelligent process management (PM) (Smart Factory) (SF) as presented by Lee et al. [11],
- the need to cope with increasing complexity of value-stream networks (VSN) as researched by Schuh et al. [12],
- the necessary paradigm shift towards strategic alignment as pointed out by Covey [13].

In the context of LF and SF, empowerment can be understood as a systematic way of learning that enables continuous improvement in an autonomous, intelligent, self-organized, and systematic manner. Coleman [14] defines empowerment as “the act of enhancing, supporting or not obstructing another’s ability to bring about outcomes that he or she seeks.” An “autonomous” management method ought to be able to function without a centralized controlling force by empowering all organizational elements. An “intelligent” management method ought to sustainably empower all organizational individuals to align and grow in the direction where value comes from for the organization.

A powerful paradigm to empower organizations focuses on value creation has flourished in the last two decades as lean management (LM). LM has been declared to be the industrial paradigm of the 21st century by Shah and Ward [15]. It is believed that the *problems* that LM endeavors to solve, the non-value adding activities, are embedded within processes, and therefore the response-able process owners (PO) that manage them are in charge of eliminating the non-value adding activities within them. Thus, the task set by LM is mainly a process management task and not a problem-solving one. Each individual of the organization is understood to be a PO, who is acting on his or her process on the shop floor.

This also enhances the need to act autonomously to make fast and flexible adjustments. Nonetheless, it is necessary to be aligned in the same direction (HOSHIN) by the strategic goals of the organizations. This leads to the fact that a balancing act between empowerment and alignment towards strategic goals is needed. According to Frow et al. [16] this makes multiple controls necessary. HOSHIN KANRI (HK) (management by giving direction) is a comprehensive management system that enables such alignment of complex systems as shown by Jolayemi [17].

Many of these points are in the general focus of SM. The term “shopfloor” has been used by western scholars, de Leeuw and van der Berg [18], to refer to processes close to production or distribution, excluding purposely strategic processes. In this sense, SM can be understood as a management system that can enhance shopfloor performance. The term “shopfloor” is used by Japanese scholars, Suzaki [19], in a broader sense, understanding “shopfloor” or “gemba” as the place, physical or virtual, where the value stream (VS) is performed. The definition of VS that Womack and Jones [20] gives is a “sequence of activities required to design, produce, and provide a specific good or service, and along which information, materials, and worth flows.”

Because of the before mentioned challenges, efficient SM systems will be essential in the transformation process towards Industry 4.0 [21]. Thus, it is of chief benefit to rate these options. Earlier papers have focused on the implementation effects of case studies [22,23,24,25] or used theoretical considerations [26]. A big detriment is the enormous effort to measure the direct influence of the management strategies. For a comparison, similar starting positions are necessary. Only then are the outcomes contrastable. The many influencing factors make it challenging to get meaningful results from only a few comparisons. So, alternative concepts that produce more comparable findings are of immense value. Examining the brain activity during the practice of the SM methods opens up unique possibilities.

Earlier research demonstrates that significant neurological variations of a PO using different LM techniques such as KATA and (CPD)nA exist [27]. The aimed added value through this paper is manifold. Instead of only looking at the brain activity of the PO, this research further takes into account the interaction with his supervisor (in the following “leader”) by recording the EEG data of both. It also concentrates on the differences of various *SM systems*. LM and SM have a large overlap, as SM uses many LM methods and tools. The primary distinction is that SM focuses on the aspects of leading and empowering people on the shopfloor [28], the place where the value creation occurs. Furthermore, an alternative way of pre-processing the EEG data for the DL soft sensor is implemented.

## 3. Literature Review

Much is still unknown about how the brain functions and above all the human brain. Despite this, many remarkable discoveries of the recent years promote the understanding [29,30]. These findings can help Industry 4.0 leaders in distinct ways. They make it easier to understand which factors are most important to foster progress on the shopfloor. This is attributed to the circumstance that the crucial component in the advancement of a company continues to be using the full potential of the employees [31].

The essential element for continuous improvement through the workers is the ability to form internal goals and to pursue these. In this context, the prefrontal cortex (PFC) has been identified as the most important region of the brain that contributes to this [32]. It is the center for cognitive control and makes it possible to act flexible to the outer world. For automatic “*hardwired*” behaviors, it doesn’t play any significant role. This leads to the conclusion that substantial activity in the PFC should be expected and seems to be a requirement for POs practicing an SM system.

Another critical factor is the cooperation between workers to achieve improvements. Understanding the mental state of others is a prerequisite. In this context, there is sound evidence that not only the PFC plays a significant task. Likewise, the left and right temporoparietal junctions (TPJ) are indispensable. Though the roles they play seem to differ. While the left appears to be involved in strategic planning of choices concerning humans [33], the right plays a pivotal role in empathy, sympathy and perception [34,35].

Next to the PFC, the right TPJ additionally seems to play an important role for attention shifting [36]. This is a fundamental aspect in an Industry 4.0 setting, as every part of a manufacturing process is intertwined with other processes. These need to be put into consideration during any change process. Hence it is necessary to be capable of moving the mental focus.

To analyze the differences between SM systems, two distinct groups can be identified by focusing on the goal achievement that lays at the core of each management system.

1. Focus on pre-defined goals
  Through pre-defined goals, the focus is set on finding ways to achieve these. This is done through specific key figures, that in the best case give a balanced view on the different achievements or KPIs [37,38,39].
2. Continuous improvement by giving directions
  This group of SM systems only provides a direction (HOSHIN) of improvement. A pre-defined goal is not set. Through this, improvements are approached in a more agile form and can be adapted along the road [40,41,42,43].

To narrow down the further analysis, one example was chosen for each of the two categories. The Balanced Scorecard (BSC) [44] as a representation of SM systems with pre-defined goals and the Hoshin Kanri TREE (HKT) representing the focus on continuous improvement by giving directions [45].

**Figure 2:**
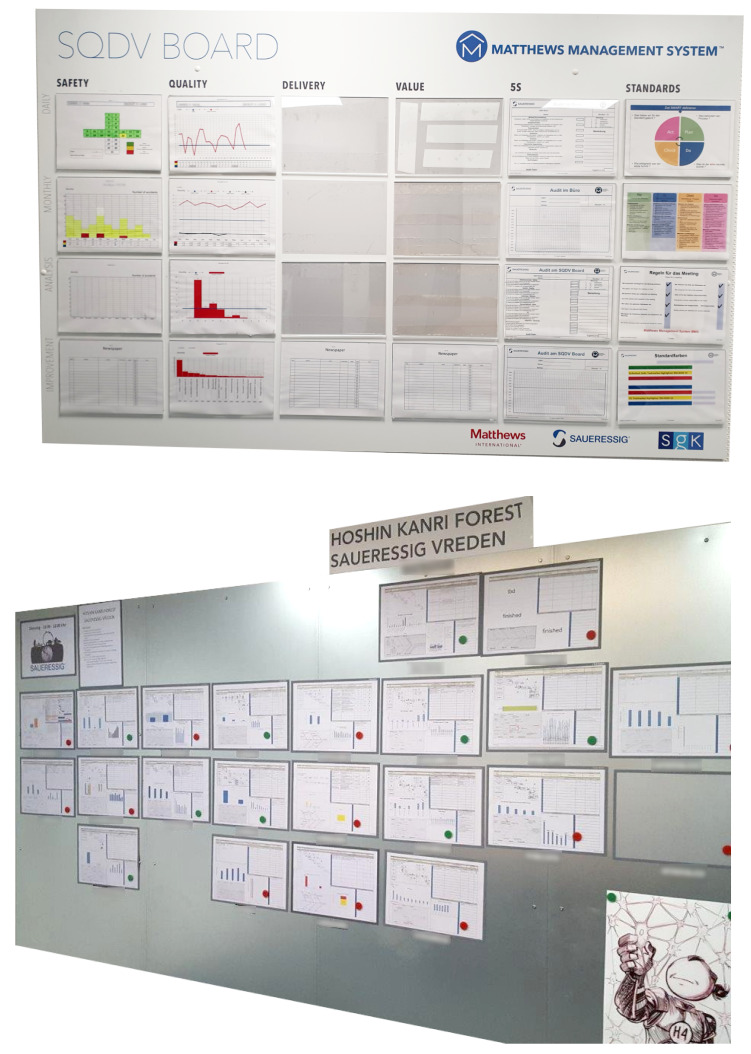
Shopfloor management systems. (**a**) SQDV (Safety, Quality, Delivery and Value) board at Matthews International GmbH, Vreden, Germany as an example of a balanced scorecard management system [44]. (**b**) Hoshin Kanri TREE [45] at Matthews International GmbH, Vreden, Germany. Process owner names are blurred out for privacy reasons.

1. *Balanced Scorecard* is a SM system first described by Kaplan et al. [44] in 1992. The prime goal is to enable a *balanced* view on the driving measures of a business. This works by showing a handful of measurements that allow managers to interpret the complex interactions. Every measurement receives a specific target to motivate the employees to achieve this state. This is in contrast to more traditional approaches. A focus was only set on a few financial performance numbers. These only give a very short-sighted glance on the actual competitiveness of a company.
  One example of a balanced scorecard implementation is visible in Figure 2a showing the measures of **S**afety, **Q**uality, **D**elivery and **V**alue (*SQDV*). There are many variants in circulation such as **S**afety, **Q**uality, **D**elivery and **C**ustomer (*SQDC*) or **Q**uality, **D**elivery, **I**nventory, and **P**roductivity (*QDIP*) that can also show the priorities of a company by including or excluding specific categories such as **E**nvironment or **S**afety.
  Neely describes the standard way of using *BSC* [46] through the following steps:
  I **Check the current performance**. See how the development is progressing.
  II **Communicate performance**. Bring everyone to the same understanding of the current state.
  III **Confirm priorities**. Align the actions needed to improve the performance.
  IV **Compel progress**. Systematically achieve better performances.
  The prime aim is to measure and communicate the achievements towards **predetermined** goals [47]. Niven summarizes balanced scorecard as a conversation tool, a measurement system and a strategic management system [48].
2. *HKT* [45] in contrast is an example of a SM system that focuses on continuous improvement without pre-defined goals. A key feature is the standardization of communication between *PO*s in organizations using the *(CPD)nA* framework. Based on this, a feedback empowerment loop is implemented which makes it possible to build a sustainable process development.
  An implementation of the *HKT* can be seen in Figure 2b. The standard procedure encompasses the following steps as described by Villalba-Diez [49]:
  I **Evaluating progress**. Every PO checks if an improvement has been made to his or her *KPI* and places either a red or green magnet on his *(CPD)nA*.
  II **Reporting progress**. Only when the *KPI* receives a red magnet, does the progress need to be announced. The others can choose.
  III **(CPD)nA**. Every reporting PO ought to follow the *(CPD)nA* behavioral pattern.
  IV **Shopfloor visit**. One of the *(CPD)nA*s improvement is checked on site by the complete team.

As shown in Figure 2, a visual representation of both types of SM systems displays the main differences between them: BSC is basically depicting a set of key performance indicators as time series, and the HKT is representing a continuous improvement focused communication network between POs.

To analyze these, an experimental setup using EEG recordings can be implemented. Depending on the results to be achieved with the EEG data, distinct methods of investigation are possible. A limited use case is the manual inspection for abnormalities in the EEG data, which can take a long time and presumes at least a basic level of domain knowledge [50]. The frequency analysis has been a more popular method that allows a wider range of applications [51,52,53].

Two distinct techniques are used in this research. These are the correlation function and a DL soft sensor.

1. Correlation Function
  The correlation function has found many use cases. It makes it feasible to classify EEG data [54], identify risk levels for developing schizophrenia [55], detect epileptic stages [56] or to classify EEG Motor imagery [57].
  With it, it is possible to determine the similarities between two signals and many application fields use it. In image processing it is for example used for template matching [58] or local image registration [59]. In geology for the location of earthquakes [60].
  The cross-correlation function works by sliding one signal along the other, calculating the product between the signals and finding the best fit [61]. Therefore, it is possible to work with time-shifted signals with the cross-correlation function.
2. Deep Learning
  In the last years DL has become a popular technique for analyzing EEG data and has been used to recognize emotions [62,63], detect Parkinson’s [64] and Alzheimer’s [65] disease, epileptic seizure prediction [66], the detection and diagnosis of seizures [67] or to decode and visualize the EEG data [68].
  An enormous advantage is that it can handle the complex EEG data with no prior domain knowledge, which allows a wider audience to work with this data. The neural network does this by *learning* the parameters to detect features from examples [69]. This has made it a popular choice for many other fields such as computer vision [70], audio processing [71] or bioinformatics [72]. The key challenge often hindering the further progress with neural networks is the limited data available. This is a prerequisite to represent a high range of input and parameters.

Our research aims to expand this approach on the characterization of complex LM shopfloor management associated behavioral patterns in an Industry 4.0 environment. In order to achieve this, this study outlines the following four research hypotheses (H) and their related LM interpretation shown in Table 1. Furthermore, as these hypotheses are based on neurophysiological expert knowledge, management needs to be provided with tools that allow a proper discernment of which behavior is followed, based only on the data. For this reason, a DL-based soft sensor is developed that is able to perform this task. The aim is to examine these with the mentioned methods.

## 4. Materials and Methods

To test the hypotheses, a case study can provide meaningful first impressions if these are valid. Still it is necessary to note that a single study cannot give clear-cut proof. In the following the scope of the research is established Section 4.1, the population and sampling is specified Section 4.2, the data collection is further described Section 4.3, as well as the data pre-processing Section 4.4, the standardization procedure Section 4.5 and the data analysis Section 4.6.

### 4.1. Scope Establishment

**Figure 3:**
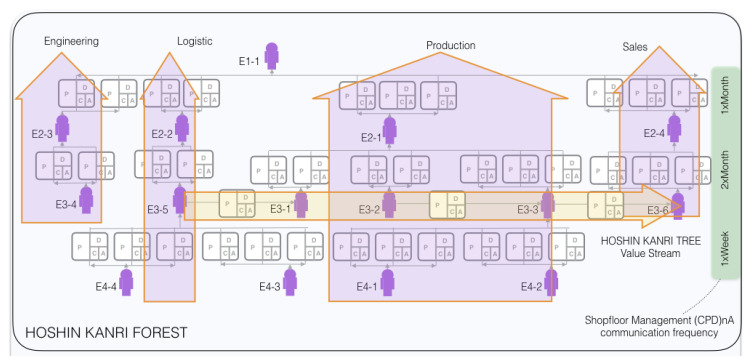
Part of the HOSHIN KANRI FOREST STRUCTURE [73].

For this study, the EEG data from a PO and his supervisor performing a *BSC* and a *HKT* implementation are recorded. All the recordings take place at one corporation. This is an automobile manufacturing facility based in Japan, where LM/SM methodologies were systematically implemented and accompanied by one author of this paper. The organization of the company can be seen through the HOSHIN KANRI FOREST STRUCTURE in Figure 3.

### 4.2. Specifications of Population and Sampling

For this paper, data was collected from 14 subject pairs comprising a PO and his supervisor. The age range is between 20 and 60 years with a mean age of 40 years. As far as possible it was made sure that no significant neurological variations between the subjects should be expected. The subjects were male and had no history of any neurological or psychiatric disorder. Neither was any on chronic medication. All participants were left-hemisphere-dominant persons. Only between the PO and the leader differences could be possible that result from different levels of SM experience. Still, these are not expected to produce significant distinctions for the EEG recordings.

### 4.3. Data Collection

To record the EEG data, following 16 channels were chosen as standardized by the American Electroencephalographic Society [74]:

[‘Fp1’, ‘Fp2’, ‘F4’, ‘Fz’, ‘F3’, ‘T7’, ‘C3’, ‘Cz’, ‘C4’, ‘T8’, ‘P4’, ‘Pz’, ‘P3’, ‘O1’, ‘Oz’, ‘O2’]

**Figure 4:**
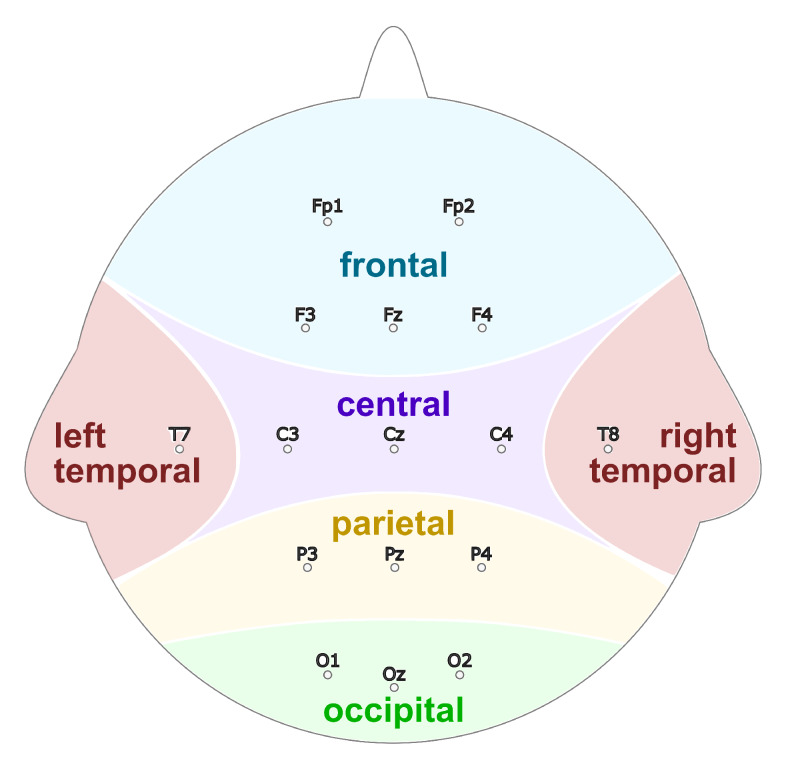
The 16 channels with standardized nomenclature that were recorded and the corresponding brain regions.

Through the regulated names, the respective locations on the head during the recording are defined. These can also be seen in Figure 4.

**Figure 5:**
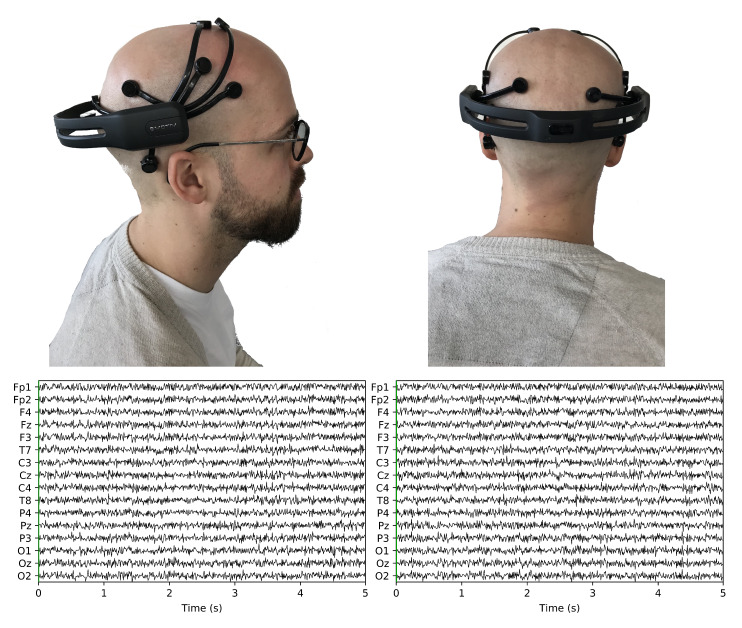
Data Collection. (**a**) EEG Low Cost Portable Sensor; (**b**) EEG Low Cost Portable Sensor; (**c**) 5 s of the recorded EEG-data for the *HKT* leader *Subject* 1; (**d**) 5 s of the recorded EEG-data for the *BSC* leader *Subject* 1.

The used EEG sensor can be seen in Figure 5a,b and has these specifications [27]:

- Sampling method: Sequential sampling. Single ADC.
- Sampling rate: 128 samples per second (2048 Hz internal).
- Resolution: 14 bits 1 least significant beat = 0.51 μV (16 bit ADC, 2 bits instrumental noise floor discarded), or 16 bits.
- Bandwidth: 0.2–43 Hz, digital notch filters at 50 Hz.
- Filtering: Built in digital 5th order Sinc filter.
- Dynamic range (input referred): 8400 μV.
- Coupling mode: AC coupled.

60 s were recorded and the hair of all subjects was cut to <1 mm to receive the best possible quality of the data.

### 4.4. Data Pre-Processing

After recording the EEG data, it needs to be pre-processed to improve the signal-to-noise ratio. This is done in multiple steps and has been achieved with ***Fieldtrip*** [75]. An open access toolbox for pre-processing and analyzing EEG data in ***MATLAB***. A high-pass filter is used to cut out the DC component of the signals, as large drifts were observed in the data. Also, a hardware embedded low-pass filter is used to remove frequencies above 50 Hz. Although it can be possible that gamma waves have an even higher frequency [76], those are usually the result of artifacts during the recording.

In the last step, the data is normalized to the range of −10 to 10, allowing the greatest level of anonymity for the probands. This doesn’t remove any essential information for the further analysis, as relative differences between subjects are not relevant for this study.

### 4.5. Standardization Procedure

For every recording, a pair of process owner and his leader are examined. For the first 10 s of the recordings, the leader talks, and the PO listens. In the remaining 50 s, the process owner presents his results. In this time, the leader observes. This is in contrast to the earlier study [27], where only the process owner of the LM system was recorded without speaking.

During the recording, the pair of PO and leader sit in a room with 50 dBA artificially created noise. This noise level is akin to that of a fridge and makes it possible to have comparable background noises.

### 4.6. Data Analysis

In the following section of this paper the developed soft sensor is further described. This encompasses the experimental setup Section 4.6.1 of the used hardware and software as well as the DL based analysis Section 4.6.3.

#### 4.6.1. Experimental Setup

The training and testing of the neural network and the pre-processing has been performed using following hardware:

- *CPU (Central Processing Unit):* Intel(R) Xeon(R) Gold 6154 CPU @ 3.00 GHz
- *GPU (Graphics Processing Unit):* NVIDIA Quadro P4000
- *RAM (Random-access Memory):* 192GB DDR4

The source code for the data pre-processing, the training, and testing of the neural network is available under Open Access Repository and was created with ***Jupyter Notebook*** Version 5.7.0.

#### 4.6.2. Correlation Function

For this paper the Pearson correlation is calculated which returns a value between −1 for a high negative correlation, 0 for no interrelationship, and +1 for a strong positive correlation [77].

#### 4.6.3. Deep Learning

After the recording and pre-processing of the EEG-data, further steps have to be taken to train the neural network.

**Figure 6:**
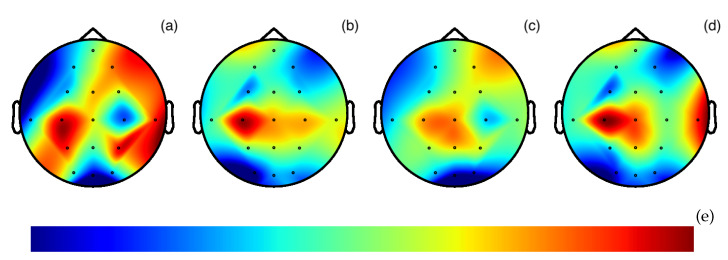
Topographic maps for the first 0.5 s of the four different categories. Each topographic map was generated from one subject. (**a**) *HKT* process owner. (**b**) *Hoshin Kanri TREE (HKT)* leader. (**c**) *Balanced Scorecard (BSC)* process owner. (**d**) *BSC* leader. (**e**) Colormap Jet used for the topographic maps.

**Figure 7:**
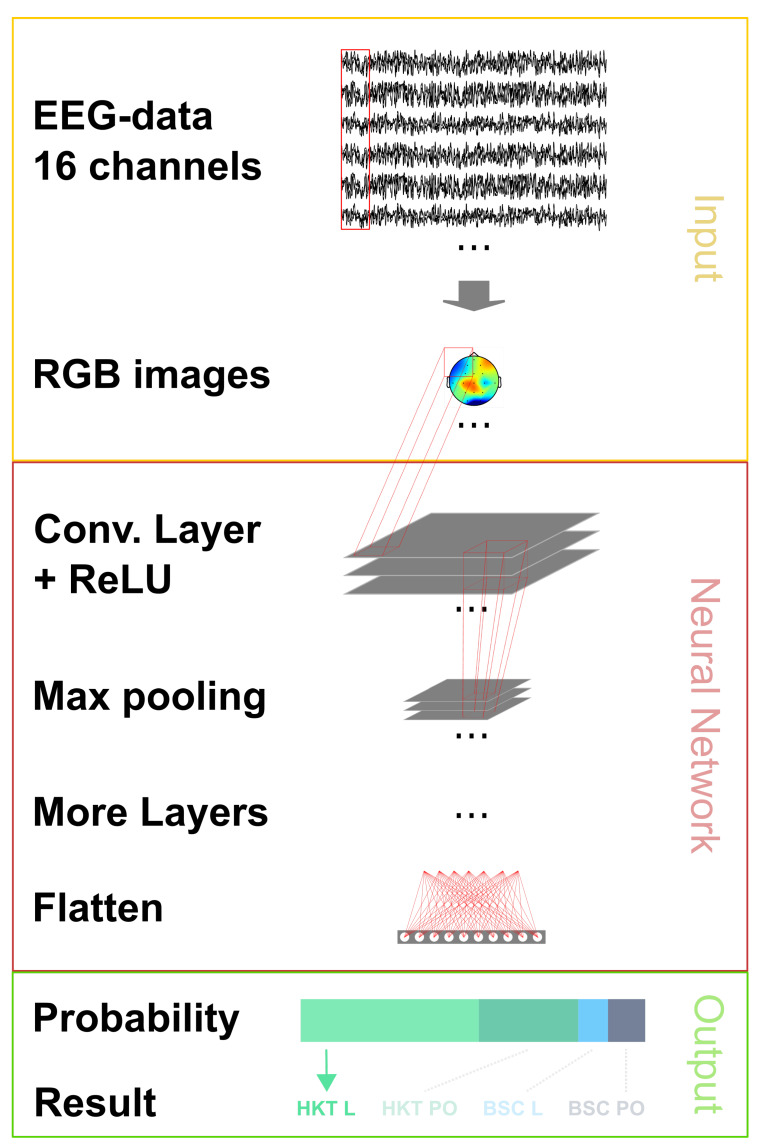
Deep Learning architecture for the classification of the EEG-data.

1. Data Segmentation
  At first the pre-processed time-dependent EEG data is split into 0.5 s long segments. It is possible to work with shorter or longer segments such as 1 s [27,78], but 0.5 s was chosen because shorter lengths would decrease the amount of information of a data point and longer lengths would decrease the amount of data points that can be used for training.
2. Image generation
  Through the MNE library [79,80], these time segments are transformed into topographic maps, as shown in Figure 6. The topographic map displays the activity in the brain, using distinct color tones for the different strengths. To show the brain activity for the complete brain and not just the measured points, the points in between the sensors are interpolated, creating a topographic map for the complete brain. Using more sensors would increase the accuracy of the interpolated area.
  In the example Figure 6 four topographic maps are visible. These show the average brain activity in the first 0.5 s for the four different categories.
  The values of the 0.5 s segments stretch to the smallest and largest value, displaying the relative differences on the brain using the *jet* color map as shown in Figure 6e. This was chosen as the full range of colors is used and the color range does not need to be optimized for the human perception as this color range is not intuitive for a human [81]. The lowest values are shown as a dark blue, which turn to a green and then to a dark red with the highest value.
  The images have a size of 360 × 360 × 3 pixel. A different size could have been chosen. Smaller images could decrease the accuracy and larger images would increase the training time.
3. Deep Learning Architecture
  With these generated images, a neural network can be trained. In this paper a convolutional neural network [82] is used. It has shown astonishing results within the realm of image classification [83,84,85] and is by far the most adopted neural network for physiological signal data processing while also showing excellent results [86]. By using it, a manual feature engineering is unnecessary. In the training phase, the neural network finds the most important features. These become more complex in each convolutional layer.
  Still, some specifications are set manually. These form the architecture. The coarse architecture of the used neural network can be seen in Figure 7, the exact composition and the code in the Open Access Repository. Parameters that are set include among others the number and the type of layers and the optimizer for the training of the neural network. Here we have chosen the *Adam* optimizer for the training of the network which stands for adaptive moment estimation [87]. The network architecture comprises four repeated layer groups consisting of a convolution, followed by a rectified linear unit (ReLU) activation and a max pooling layer. After this, the network is flattened and a regular, deeply connected neural network layer follows. To reduce the chance of over-fitting a dropout layer with a ratio of 0.2 is added. The network ends with four outputs that go through the Softmax function to display the probability of the four examined categories.
  While statistical ways exist to help select some parameters [88,89] and few have become unofficial standards, no clear-cut way to know the best beforehand exist yet. Thus, it is an iterative process of finding the ideal specifications for the architecture by starting with a simple design and seeing which changes improve the results. The final optimal layout therefore depends on multiple factors. More complex features that have to be found in general increase the number of layers needed [90]. But these can also be limited because the training data wouldn’t be sufficient for the increasing number of weights or a limit is reached solely because the hardware requirements can’t be met.

## 5. Results and Discussion

This section presents the obtained results using the recorded EEG-data. It comprises two major parts. Through the analysis of the EEG-data with the help of the correlation function, and through the analysis of the EEG-data with the help of the DL soft sensor. The real-world implications of these findings follow in Section 6.

### 5.1. Results and Discussion of the Correlation Function

The correlation function can verify the hypotheses presented in Table 1. In the following, the results for each hypothesis are examined.

**Figure 8:**
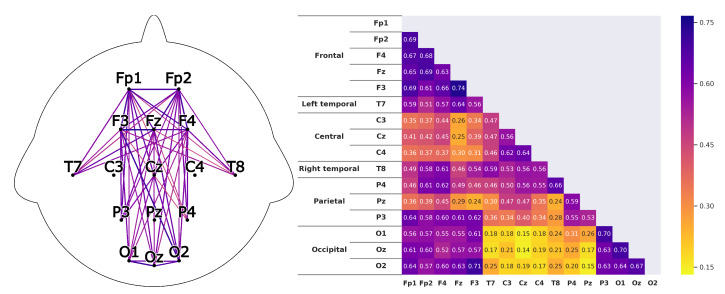
Average correlations of the EEG sensors for the *HKT* process owner displayed in two differing layouts. On the left through connecting lines. The strength is visible through the color and the thickness of the line. Only the correlations concerning the 5 hypotheses are shown to have a clearer view. On the right, the correlation matrix for all EEG sensors is displayed.

**Figure 9:**
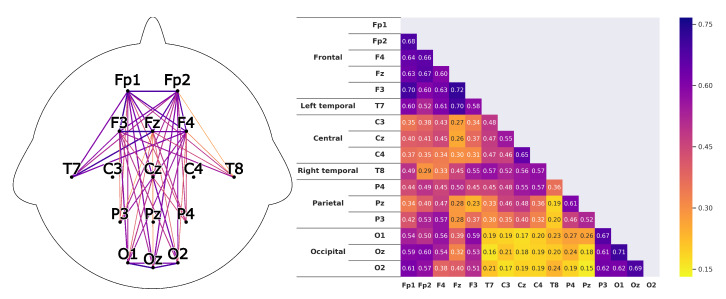
Correlations as described in Figure 8 for the *BSC* leader.

**Figure 10:**
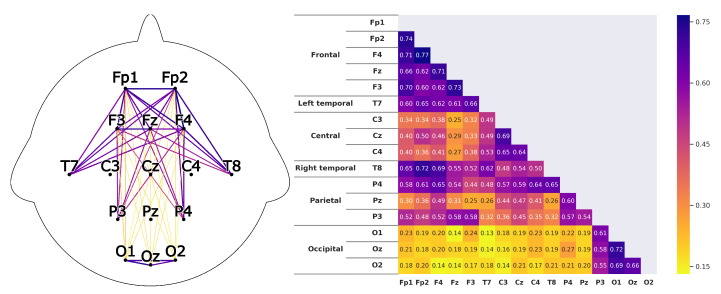
Correlations as described in Figure 8 for the *HKT* leader.

**Figure 11:**
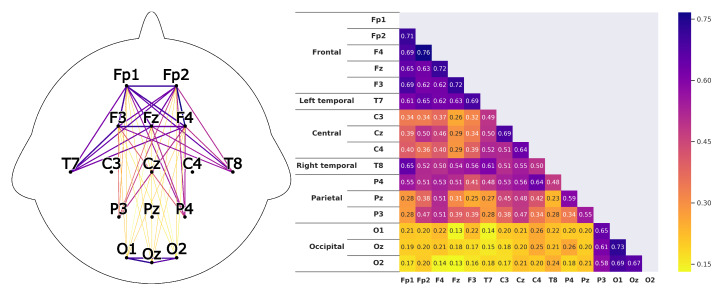
Correlations as described in Figure 8 for the *BSC* process owner.

**Figure 12:**
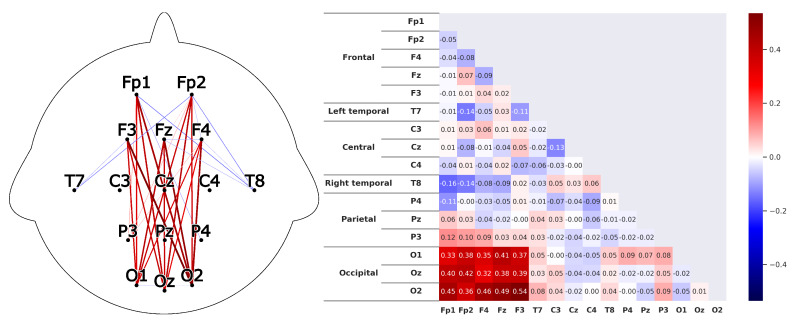
Difference of the results between the HKT leader and the HKT process owner as described in Figure 10.

**Figure 13:**
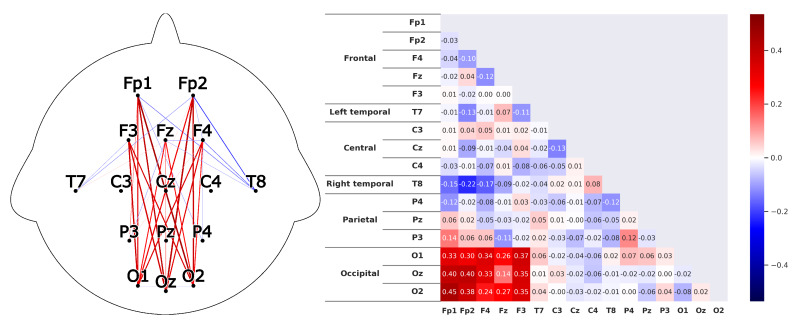
Difference of the results between the BSC leader and the BSC process owner as described in Figure 10.

**Figure 14:**
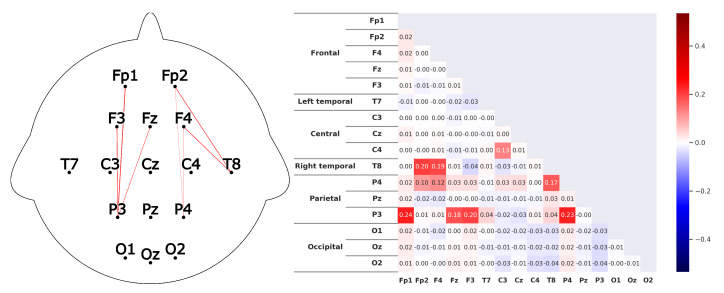
Difference of the results between the HKT process owner and the BSC process owner as described in Figure 10. Colorscale maximum and minimum are set to the +maximum and -maximum absolute value of the examined differences.

**Figure 15:**
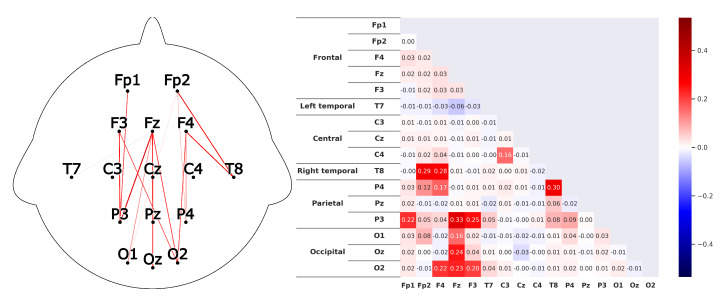
Difference of the results between the HKT leader and the BSC leader as described in Figure 10.

1. Corresponding to *H1*
  A subject that is listening shows strong correlations between the prefrontal-cortex and the occipital cortex. For the recordings this would mean that a high correlation between the sensors *Fp1-F3-Fp2-F4-Fz* and *O1-Oz-O2* can be found.
  This can clearly be seen in the Figure 8 and Figure 9. The leaders for both *HKT* and *BSC* show a strong correlation between the prefrontal-cortex and the occipital cortex with values ranging between 0.52 and 0.71 for the HKT leader and values between 0.32 and 0.61 for the BSC leader.
2. Corresponding to *H2*
  A subject that is listening shows significant correlations **within** the prefrontal-cortex and the occipital cortex. This would be seen in the recordings through high correlations within the sensor groups *Fp1-F3-Fp2-F4-Fz* and *O1-Oz-O2*.
  This is confirmed in Figure 10 and Figure 11. Both *HKT* and *BSC* process owner show high correlations within the sensor groups but not between the sensor groups. The values for the frontal sensor group are in the range from 0.6 to 0.74 for the HKT process owner and 0.62 to 0.76 for the BSC process owner. For the occipital group, the values range between 0.55 and 0.72 for the HKT process owner and 0.58 to 0.73 for the BSC process owner. The values between the frontal and occipital sensor groups only reach up to 0.23 for both HKT PO and BSC PO.
  A clear difference between the leaders and process owners can be seen in Figure 12 and Figure 13, where the biggest differences are the connections between the frontal and occipital group.
3. Corresponding to *H3*
  To confirm an executive behavioural pattern in the neurological activity, a strong correlation in the PFC should be found. For the recordings this would mean that a high correlation between the sensors *Fp1-F3-Fp2-F4-Fz* should be expected in all four examined categories.
  This is confirmed in Figure 9 and Figure 10 showing very high correlations within the frontal sensor group. The minimum value for the weakest correlation of the four subject groups still reaching 0.6, which can already be considered a strong correlation.
4. Corresponding to *H4*
  To conclude hypothesis 4, it is anticipated that strong correlations can be found between the PFC and the TPJ for *HKT* practitioners.
  In the *EEG* recordings this would be confirmed, if strong correlations between the sensors *Fp1-F3-Fp2-F4-Fz* and *T7-T8*, as well as *P3-P4* can be found.
  This can be seen in Figure 8 and Figure 10. Values range between 0.52 to 0.72 for the HKT process owner and 0.46 to 0.64 for the HKT leader.
5. Corresponding to *H5*
  To conclude hypothesis 5, it is anticipated that only weak correlations can be found between the prefrontal-cortex and the TPJ for *BSC* practitioners.
  In the *EEG* recordings this would be confirmed, if weak correlations between the sensors *Fp1-F3-Fp2-F4-Fz* and *T7-T8* as well as *P3-P4* can be found.
  The results for this hypothesis aren’t as clear-cut as the previous. Although clearly lower correlations such as 0.28 for the correlation *Fp1_P3* of the BSC PO can be found, *Fp1_T8* for the same category also shows a correlation of 0.65 in Figure 11. What can clearly be seen in the comparison of the BSC PO and the HKT PO in Figure 14, is that a handful of the expected correlations of the BSC PO show a significantly lower value compared to the HKT PO. These are the correlations *Fp1_P3*, *Fp2_T8*, *F4_T8*, *Fz_P3*, and *F3_P3*, that on average show a correlation with 0.2 less. This could mean that the right TPJ is less active for the BSC PO. Which in turn would signify that the BSC PO shows weaker activity in the brain region responsible for empathy or more general for the ability to switch between perspectives.
  The difference between the HKT leader and BSC leader in Figure 15 shows similar results as between HKT PO and BSC PO. This means that the right TPJ is also more active for the HKT leader than for the BSC leader, which would again mean that also the leader of the BSC shows less activity in the brain region linked to the ability to switch between perspectives.

### 5.2. Results and Discussion of Deep Learning Soft Sensor

With the help of the DL soft sensor, a classification rate accuracy of 96.5% was able to be achieved. This proves that an automatic classification of the EEG data without the need of any domain knowledge is possible. Furthermore, it reveals that the differences of the neurological activity of the subjects practicing either SM system are significant enough to make this achievable.

To train the DNN, the recorded EEG data was split into three parts. The training, validation, and testing set. The set size ratio depends on the amount of data, as the test and validation data should be sufficient to check the validity. While big data cases with millions of examples allow for splits of 99.5% training data, 0.25% validation data and 0.25% test data, cases with examples in the range of 10,000 need a bigger split for the test and validation data [91]. For smaller data collections the standard is a 80%, 10% and 10% split. By keeping all of the time slices of a subject pair in one split, it can be assured that the DNN does not learn specifics from the particular pass. Therefore, a split of 10/14≈72%, 2/14≈14% and 2/14≈14% was chosen. With 0.5 s slices this equates to 4800 images for the training set, 960 images for the validation set and 960 images for the test set. Though it would be possible to perform a k-fold cross-validation, this was not chosen, as the data set is big enough for a simple train/test/validation split and it would increase the training time significantly.

**Figure 16:**
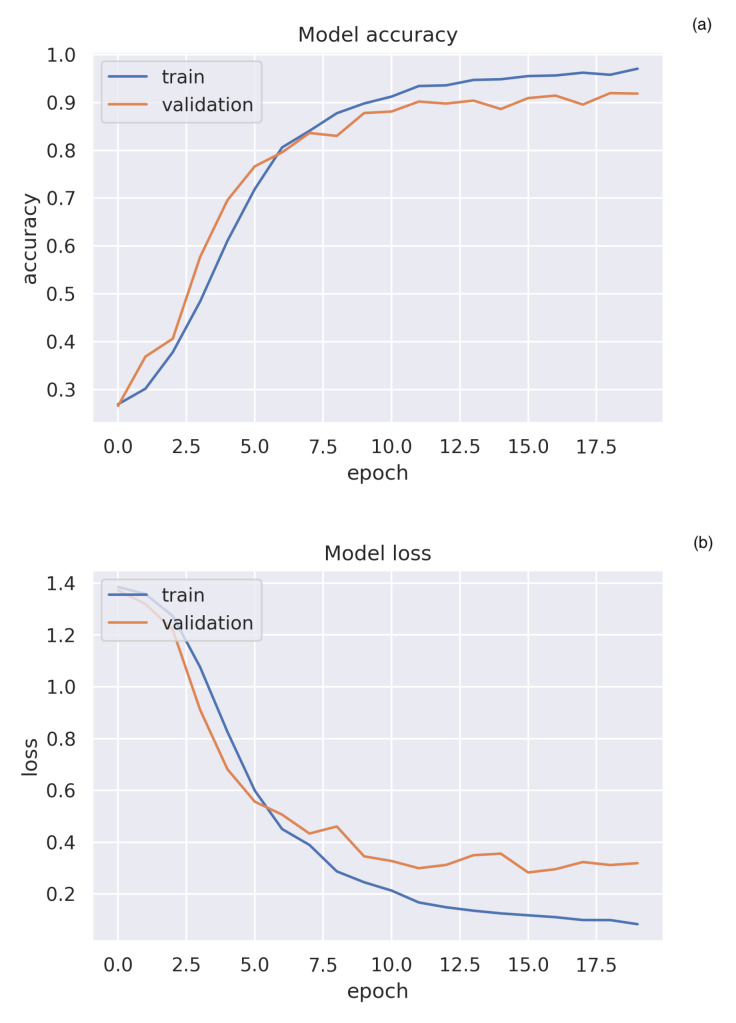
Results for the training of the deep learning (DL) network. (**a**) Training and validation loss of the trained DL network. (**b**) Training and validation accuracy of the trained DL network.

After training the DNN with the **training data** in multiple epochs (Figure 16), the **validation data** can be used to tune the hyperparameters of the DNN without exposing the **test data** to the DNN. Only at the very end, when no further changes to the DNN are being done, can the testing data be used to evaluate its effectiveness. A common method is to use the results of a confusion matrix [92]. These are usually separated into the categories True Negative, False Negative, True Positive and False Positive. As this is a multiclass categorization, it makes sense to have a True and False version for each of the four categories.

1. True *HKT PO* stands for the EEG data of POs practicing the *HKT* system, that has **correctly** been classified as a PO practicing *HKT*.
2. False *HKT PO* stands for all other EEG data, that has **falsely** been classified as a *HKT PO*.
3. True *HKT Leader* stands for the EEG data of Leaders practicing the *HKT* system, that has **correctly** been classified as a leader practicing *HKT*.
4. False *HKT Leader* stands for all other EEG data, that has **falsely** been classified as a *HKT Leader*.
5. True *BSC PO* stands for the EEG data of POs practicing the *BSC* system, that has **correctly** been classified as a PO practicing *BSC*.
6. False *BSC PO* stands for all other EEG data, that has **falsely** been classified as a *BSC PO*.
7. True *BSC Leader* stands for the EEG data of Leaders practicing the *BSC* system, that has **correctly** been classified as a leader practicing *BSC*.
8. False *BSC Leader* stands for all other EEG data, that has **falsely** been classified as a *BSC Leader*.

**Figure 17:**
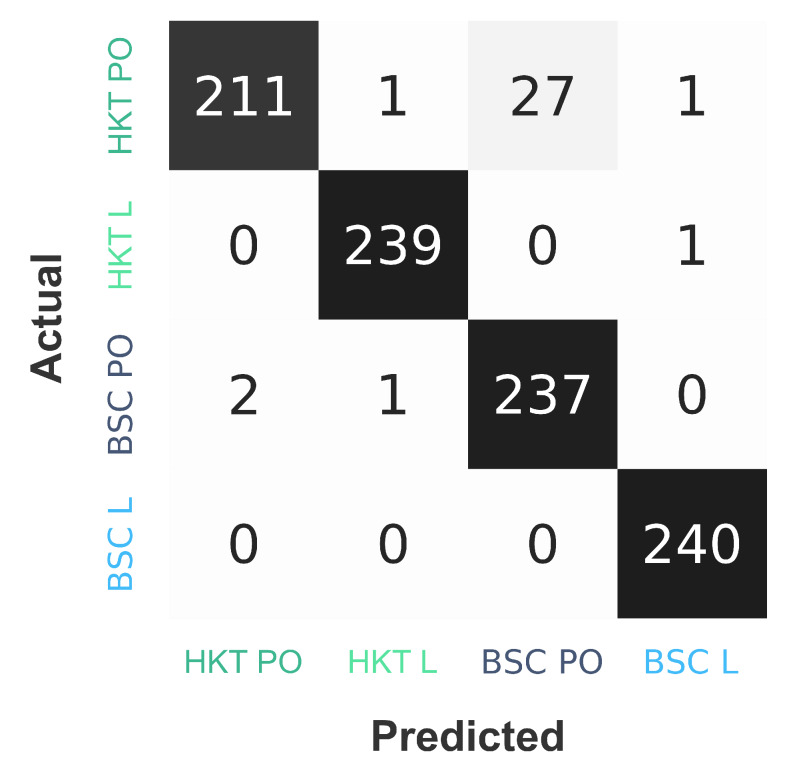
Multi-label confusion matrix (n = 960) without normalization for the four categories that have been used to train the neural network.

The results from the confusion matrix can be seen in Figure 17. With these values, further values for the evaluation of the model can be computed. The accuracy of the DNN can be calculated by dividing the sum of the True cases through the sum of all cases: (THKTPO+THKTL+TBSCPO+TBSCL)/(THKTPO+THKTL+TBSCPO+TBSCL+FHKTPO+FHKTL+FBSCPO+FBSCL). In this study 96.5% has been achieved, which is extremely high, as EEG data usually has a poor signal-to-noise ratio and significant differences between subjects should be expected [93]. Other studies using EEG data and DL for the classification of brain data have also shown positive results. Though it is difficult to compare the applied classification algorithms, as there are few public data sets that are consistently used to evaluate the effectiveness of these. Next to CNN, Recurrent Neural Networks (RNN)/Long-short Term Memory (LSTM) implementations have been used extensively. Depending on the classification type, accuracies up to 100% could be achieved [86].

## 6. Management Conclusions and Future Steps

This paper uses a multidisciplinary approach to find ways that can help Industry 4.0 leaders guide the shopfloor. As it forms the center of the value-creation a specific focus needs to be laid on it. Through wide-ranging fields such as neuroscience, machine learning and management science, it has been possible to discover various interesting aspects that support interpreting the thinking processes during the practice of SM systems. It requires to be noted that these are just starting points that need to be confirmed in further studies. The understanding of the brain regions functions is ongoing and the ability of the regions are not limited to one specific function. The interplay is even more complex and sometimes allows for a wide range of explanations.

Still, following interpretations can be drawn from the achieved results:

- The studied SM systems both cause large correlations within the PFC for the PO and the leader. This indicates that both systems and both PO and leader show brain activity that can be considered goal-oriented, which is the core requirement for any kind of improvement.
- Both BSC and HKT show a solid correlation between the PFC and the left TPJ. The left TPJ amongst other functions plays an important role in the strategic planning concerning other people. The difference between BSC and HKT is negligible, but the difference between PO and leader show interesting patterns. Both correlations *T7_Fp2* and *T7_F3* show noticeable stronger results for the PO. This arises from the different focuses of PO and leader.
- The HKT PO shows substantial correlations of the PFC to the right TPJ. This is especially noticeable in comparison to the results of the BSC participants. The direction-focused approach seems to enable a wider view, allowing diverse positions to be taken into perspective. This is a crucial characteristic for an Industry 4.0 setting where conflicting information needs to be taken into consideration to find optimal improvements that not only shift the focus but ensure sustainable improvements [94]. The HKT leader shows significantly weaker results in this aspect.
- The SM system BSC on the contrary demonstrates weak correlations for the right TPJ to the PFC for both PO and leader, while the leader also shows significantly weaker correlations. The reduced correlations of the PFC to the right TPJ indicate that the contemplated perspectives are restricted and the focus on pre-defined goals limits the aspects that are taken into consideration to receive a goal.

Although the results can be described as preliminary, due in part to the relatively small sample space, if confirmed, the conclusions of this study could have profound implications in the world of Western management. The fundamental reason for this assertion lies basically in the fact that practically the entire corporate culture of operational management is based on the achievement of objectives. In a business environment in which the complexity of processes is constantly increasing, management models that favour a holistic understanding of this reality. Studies such as the one presented, which is clearly multidisciplinary, present, with the help of artificial intelligence, the differences between types of management and the effects that management could be having on POs in a quantitative way.

The classification results of the EEG-data emphasize that significant and distinct differences in the brain activity of the examined leadership patterns exist and can be learned by a DNN. Furthermore, the implemented classifier allows for different usages. As the classification can be done in real time, direct feedback can be given. This could, for example, be used during the training of an SM system. An employee with an EEG-device connected to the trained DNN could receive valuable feedback to differentiate the systems and better understand the thinking patterns.

A comparison of the subjects would be of high interest in order to know how far these results are valid for all subjects and which differences can expected. In this way possible differences between experience, age and gender could be examined and it would be possible to see if alterations in the group size could possibly change the results. Due to compliance rules of the organization this was not possible.

Future research could test the influence of the cultural background and how experience in LM/SM changes the subjects brain patterns. It could also be of interest to examine the influence of the subject speaking on the results.

## Figures and Tables

**Table 1:** Hypotheses regarding the correlations of the recorded electroencephalography (EEG)-data.

#	Hypothesis	Interpretation
H1	The brain patterns of the leaders are expected to show strong correlations between the prefrontal-cortex and the occipital-cortex. This causes high correlations between the sensors *Fp1-Fp2-F3-Fz-F4* and *O1-Oz-O2*.	This result can be expected, as the leaders are listening for the majority of the time.
H2	In contrast to *H1*, the brain patterns of the process owners are expected to show significant correlations within the prefrontal-cortex and the occipital-cortex. This could be seen in strong correlations within the sensor groups *Fp1-Fp2-F3-Fz-F4* and *O1-Oz-O2*.	This result can be expected, as the process owner speaks for the majority of the time.
H3	Besides *H2*, all subjects are expected to show a high correlation of the prefrontal-cortex. This causes high correlations for the sensors *Fp1-Fp2-F3-Fz-F4*.	This could be understood in that way, that the conducted tasks are all executive behavioural patterns.
H4	The brain patterns of *HKT* practitioners are expected to show a strong correlation between the prefrontal-cortex and the TPJ. This could be seen by high correlations between the sensors *Fp1-Fp2-F3-Fz-F4* and *T7-T8* as well as *P3-P4*.	The interpretation is that *HKT* is a goal-oriented, context-independent *SM* problem-solving behavioral pattern.
H5	Compared to *HKT*, the brain patterns of *BSC* practitioners are expected to show a weak correlation between the prefrontal-cortex and the TPJ. This could be seen by low correlations between the sensors *Fp1-Fp2-F3-Fz-F4* and *T7-T8* and *P3-P4*.	This could be understood in that way, that *BSC* is a goal-oriented, context-dependent *SM* problem-solving behavioral pattern.

## Abbreviations

The following abbreviations are used in this manuscript:

BSC	Balanced Scorecard
(CPD)nA	Check-Plan-Do-…-Act
dBA	Decibel A-weighting
DL	Deep Learning
DNN	Deep Neural Network
EEG	Electroencephalography
HK	HOSHIN KANRI
HKT	HOSHIN KANRI TREE
KPI	Key Performance Indicator
LM	Lean Management
PDCA	Plan-Do-Check-Act
PFC	Prefrontal Cortex
PO	Process Owner
SM	Shopfloor Management
TPJ	Temporoparietal junction
VS	Value Stream
